# Comparative Transcriptomic and Expression Profiles Between the Foot Muscle and Mantle Tissues in the Giant Triton Snail *Charonia tritonis*

**DOI:** 10.3389/fphys.2021.632518

**Published:** 2021-02-24

**Authors:** Gege Zhang, Meng Xu, Chenglong Zhang, Huixia Jia, Hua Zhang, Maoxian He, Wenguang Liu

**Affiliations:** ^1^CAS Key Laboratory of Tropical Marine Bio-Resources and Ecology, Guangdong Provincial Key Laboratory of Applied Marine Biology, South China Sea Institute of Oceanology, Chinese Academy of Sciences, Guangzhou, China; ^2^University of Chinese Academy of Sciences, Beijing, China; ^3^Southern Marine Science and Engineering Guangdong Laboratory, Guangzhou, China; ^4^Sansha Marine and Fisheries Bureau, Sansha, China

**Keywords:** biomineralization, transcriptome, *Charonia tritonis*, foot muscle, mantle tissue

## Abstract

The giant triton snail (*Charonia tritonis)*, an endangered gastropod species of ecological and economic importance, is widely distributed in coral reef ecosystems of the Indo-West Pacific region and the tropical waters of the South China Sea. Limited research on molecular mechanisms can be conducted because the complete genomic information on this species is unavailable. Hence, we performed transcriptome sequencing of the *C. tritonis* foot muscle and mantle using the Illumina HiSeq sequencing platform. In 109,722 unigenes, we detected 7,994 (3,196 up-regulated and 4,798 down-regulated) differentially expressed genes (DEGs) from the *C. tritonis* foot muscle and mantle transcriptomes. These DEGs will provide valuable resources to improve the understanding of molecular mechanisms involved in biomineralization of *C. tritonis.* In the Gene Ontology (GO) database, DEGs were clustered into three main categories (biological processes, molecular functions, and cellular components) and were involved in 50 functional subcategories. The top 20 GO terms in the molecular function category included sulfotransferase activity, transferring sulfur-containing groups, and calcium ion binding, which are terms considered to be related to biomineralization. In KEGG classifications, transcriptomic DEGs were mainly enriched in glycosaminoglycan biosynthesis-chondroitin sulfate/dermatan sulfate, and sulfur metabolism pathway, which may be related to biomineralization. The results of qPCR showed that three of the eight genes examined were significantly up-regulated in the mantle. The phylogenetic tree of BMP1 suggested a significant divergence between homologous genes in *C. tritonis*. Our results improve the understanding of biomineralization in *C. tritonis* and provide fundamental transcriptome information to study other molecular mechanisms such as reproduction.

## Introduction

The giant triton snail (*Charonia tritonis)* is an ecologically and commercially important species that belongs to Mollusca, Gastropoda, Ranellidae, and mainly inhabits in the sandy bottom of coral reefs in the Indo-West Pacific region, as well as the tropical waters of the South China Sea (Beu, 1970; Aungtonya, 1994). *C. tritonis* preys on echinoderms especially the crown-of-thorns starfish (COTS, *Acanthaster planci*), which is considered to have importantly potential outcome on the biodiversity of coral reefs and their biological communities (Morton, 2012; Doxa et al., 2013a,b; Kang et al., 2016). As this mollusk is highly valued for its decorative shell, overfishing has driven this organism near to extinction in most of its area of habitats (Zhang et al., 2013). Despite the important ecological value of *C. tritonis*, basic information on this organism is very limited (Hall et al., 2017).

Some preliminary studies of *C. tritonis* have been proceeded in connection with the growth, reproduction processes and egg-laying patterns, as well as analysis in development and morphological of early life history stages, feeding behavior in captivity, and the important to limit population outbreaks of COTS in environments (Zhang et al., 2013). The next-generation transcriptome sequencing and *in silico* protein analysis of salivary glands were firstly reported by Bose et al. (2017b), and identified numerous secreted proteins including putative toxin- and feeding- related protein families (Bose et al., 2017a). And a cerebral ganglia transcriptome of *C. tritonis* were sequenced using Illumina HiSeq 2500 sequencing platform, with an emphasis on the bioinformatic analysis of neuropeptide genes with mollusk reproduction (Bose et al., 2017b). However, to date, no studies on biomineralization genes and pathways have been reported in *C. tritonis*. Mollusk shells are an exoskeleton mainly constituted of a large set of calcium carbonate and organic macromolecules, including proteins, glycoproteins, polysaccharides and sometimes lipids, which are secreted by mantle tissue during the biomineralization process. Numerous biomineralizing genes were found to be responsible for shell formation in various molluscan species, such as genome and multi-omic analysis of biomineralization in *Pinctada fucata martensii*, genomic databases and proteomic in-depth analysis of shell matrix construction-related genes in the limpet *Lottia gigantea*, transcriptome analysis for potential biomineralization-related genes in *Tectus pyramis*. The next-generation sequencing technology has been significantly applied to non-model organisms because it is efficient and cost-effective, which provided an efficient way to identify putatively functional genes and explain molecular mechanisms involved in the processes of biomineralization in mollusks (Moreira et al., 2015; Shi et al., 2018, 2019).

In this study, we first performed *de novo* transcriptome sequencing of the foot muscle and mantle tissues of *C. tritonis* using the Illumina sequence platform, which will provide transcriptome resources for this mollusk. Additionally, the comparison with foot muscle and mantle tissue transcription in *C. tritonis* allows to investigate and identify the biomineralization-related genes and pathway at molecular level, so as to provide further insight into molecular mechanisms involved in biomineralization for mollusk.

## Materials and Methods

### Sample Collection

The adult *C. tritonis* were obtained from Nansha archipelagic waters of the South China Sea. Tissue samples (foot muscle and mantle) were separated and quickly frozen in liquid nitrogen for 24 h, and then stored at −80°C until total RNA extraction.

### Total RNA Extraction, Illumina Sequencing, and Library Construction

Total RNA was isolated from each sample of *C. tritonis* using a Mollusk RNA kit (Omega, Atlanta, GA, United States) according to the manufacturer’s instructions. RNA integrity was detected by 1% agarose gel and the purity of the RNA was identified by using NanoDrop (Thermo Fisher Scientific, Waltham, MA, United States). Then, the total RNA concentration, the RNA integrity number (RIN), 28S/18S, and the fragment length distribution were measured using Agilent 2100 Bioanalyzer and Agilent RNA 6000 Nano Kit, (Agilent Technologies, CA, United States). The sample mRNA was enriched from total RNA using the oligo (dT) method and fragmented using a suitable reagent under high temperature. First-strand cDNA was synthesized using fragmented mRNA as a template. The reaction system was then configured to synthesize two-strand cDNA. The cDNA fragments were purified using purification kits and resolved with elution buffer for end reparation and single nucleotide A (adenine) addition. The cDNA fragments were connected to adapters and those of a suitable size were selected as templates for PCR amplification. The quality of the library constructed with PCR products was assessed by an Agilent 2100 Technologies Bioanalyzer (Santa Clara, CA, United States) and the ABI StepOnePlus Real-Time PCR System (Foster City, CA, United States). High-throughput sequencing was performed using Illumina HiSeq 2000 Technology at BGI Shenzhen (Shenzhen, China).

### *De novo* Assembly and Functional Annotation

Clean reads were obtained from raw reads by removing adaptor-polluted reads, reads with unknown bases (N) at more than 5%, and low-quality reads. High-quality clean reads were *de novo* assembled to transcripts using Trinity software (v2.0.6). Transcripts were clustered to unigenes with Tgicl software (v2.0.6). Unigenes obtained from each sample were clustered again using Tgicl, and the final unigenes for downstream analysis were named “All-Unigenes.” Unigenes were aligned to the NCBI non-redundant protein (NR), NCBI nucleotide (NT), Clusters euKaryotic Ortholog Groups (KOG), Kyoto Encyclopedia of Genes and Genomes (KEGG), and SwissProt databases to conduct functional annotation using BLAST^1^ (E-value ≤ 1e^–5^). InterProScan5 (v5.11-51.0) was used to obtain InterPro annotations. Unigenes with NR annotations were further analyzed with Blast2GO (v2.5.0) to obtain their Gene Ontology (GO) annotations.

### Analysis of Functional Enrichment of Differentially Expressed Genes

The clean reads of each sample were mapped to unigenes with Bowtie2 software (v2.2.5) and the gene expression levels were calculated using RSEM (v1.2.12). Fragments per kilobase of transcript per million (FPKM) mapped reads were used to evaluate the expression level of unigenes based on read counts and transcript lengths. Differentially expressed genes (DEGs) were detected by the PossionDis algorithm based on comparing the FPKM mapped reads between samples with thresholds of | log2FC| ≥ 2 and false discovery rate (FDR) ≤ 0.001. All identified DEGs were mapped to GO and KEGG annotations to classify. GO and KEGG enrichment analyses were performed with the phyper package in R. The *p*-value was calculated in a hypergeometric test and then the *p*-value results were adjusted based on the FDR. *p* < 0.05 was considered significant (^∗^), and *p* < 0.01 was considered highly significant (^∗∗^).

### Screening of Transcripts Involved in Biomineralization

Based on the functional annotation of DEGs in the mantle transcriptome of *C. tritonis* and the representative biomineralization genes in the mantle of mollusks published in the database, a total of 24 putative biomineralization-related transcripts were screened by searching BLASTx.

### Validation of DEGs by Quantitative Real-Time PCR

Quantitative real-time RT-PCR (qRT-PCR) was performed using 24 DEGs annotated as biomineralization-related genes in the mantle and foot muscle tissues from three *C. tritonis*. Tissues were ground in a homogenizer (IKA, Staufen, Germany). Total RNA was extracted from the foot muscles and mantles of mature *C. tritonis* (three individuals from each tissue) using the TRIzol reagent (Invitrogen, Carlsbad, CA, United States), after which the quality was checked as described above and then a ReverTra Ace qPCR RT master mix was used with a gDNA Remover kit (Toyobo, Japan) to synthesize cDNA template. The primers were designed by the software Primer Premier version 6.0^2^. Primers for amplification of target genes and the 18SrRNA internal reference gene are listed in Supplementary Table 1. The qRT-PCR analysis was conducted by using a Roche LightCycler 480 Real-time PCR System (Roche, Switzerland) with SYBR Green Real-time PCR Mix (Toyobo, Japan) according to the manufacturer’s protocols and using the following reaction conditions: 95°C for 10 s, followed by 50 cycles of 95°C for 10 s, 57°C for 15 s, and 72°C for 15 s. At the end of the reaction, a melting curve was generated to confirm an accurate amplification of the target and the reference gene 18S amplicon. Crossing point (Cp) was recorded to calculate the relative expression of target genes using the 2^–ΔΔCt^ transformation. All results are expressed as the mean ± SD. The SPSS 23.0 (SPSS, Chicago, IL, United States) was used for statistical analysis. A *p*-value of <0.05 indicated a significant difference and *p* < 0.01 indicated a highly significant difference.

## Results

### Illumina Sequencing and *de novo* Assembly

A total of 13.23 Gb raw sequencing data were generated through the Illumina HiSeq 2000 platform. After filtering, 44.07 M clean reads with a total of 6.61 Gb clean bases were acquired with 92.83% Q30 bases from *C. tritonis* foot muscle, and the 44.12 M clean reads with a total of 6.62 Gb clean bases were acquired with 92.79% Q30 bases for *C. tritonis* mantle (Table 1).

**TABLE 1:** Clean read quality metrics.

	*Charonia tritonis*	*Charonia tritonis*
	foot muscle	mantle
Total raw reads (M)	45.34	45.34
Total clean reads (M)	44.07	44.12
Total clean bases (Gb)	6.61	6.62
Q20 (%)	96.84	96.86
Q30 (%)	92.83	92.79
Clean reads ratio (%)	97.20	97.30

Based on high-quality clean reads, 183,823 transcripts were assembled with an average length of 419 bp from the *C. tritonis* foot muscle data, and 168,326 transcripts were assembled with an average length of 430 bp from the *C. tritonis* mantle data. After clustering, 87,443 unigenes with a mean length of 419 bp and an N50 of 476 bp were generated from the foot muscle. A total of 81,100 unigenes were assembled from the mantle, with a mean length of 597 bp and N50 of 843 bp. The GC percentage of the unigenes was 43.26% in the foot muscle and 43.78% in the mantle. The unigenes of these two tissues were clustered using Tgicl and the final unigenes for downstream analyses were named “All-Unigenes.” The length distribution of All-Unigenes is shown in (Supplementary Figure 1). The above results show that the quality of transcriptome sequencing and assembly was credible to subsequent analysis.

### Functional Annotation

**FIGURE 1:**
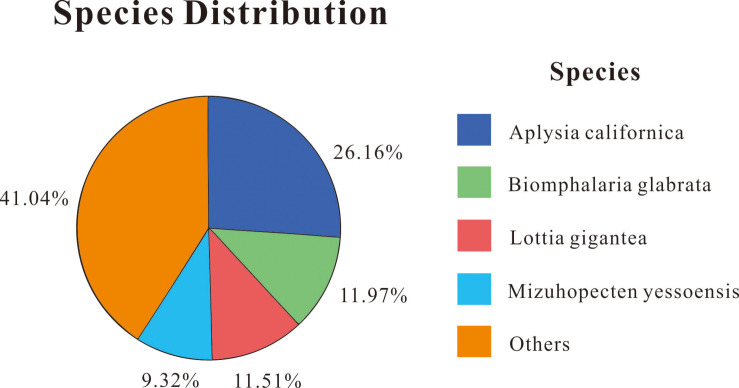
Distribution of NR annotated species.

Out of 109,722 unigenes, 46,239 (42.14%) obtained annotations in at least one public database, which included NR, NT, SwissProt, KEGG, KOG, InterPro, and GO, using BLAST (E-value ≤ 1 × 10^–5^). The most unigenes were annotated in the NT database (27,059, 24.66%), followed by NR (26,050, 23.74%), KEGG (18,705, 17.05%), InterPro (17,617, 16.06%), Swiss-Prot (16,944, 15.44%), KOG (15,319, 13.96%), and GO (9,788, 8.92%). Based on the NR database function annotation results, the best match of the species distribution for each sequence is shown in Figure 1. Among these annotated species, the largest number of unigenes matched with *Aplysia californica* (26.16%), followed by *Biomphalaria glabrata* (11.97%), *Lottia gigantean* (11.51%), and *Mizuhopecten yessoensis* (9.32%). In addition, 41.04% unigene sequences could not be matched to known genes (Figure 1).

According to GO annotation, 9,788 unigenes were categorized into 58 GO terms in the biological process, cellular component, and molecular function ontologies. In the biological process category, most of the unigenes were clustered into 26 classifications. The categories that contained the most annotated genes were “cellular process” (3,682, 27.88%), “metabolic process” (2,613, 19.79%), and “biological regulation” (1,358, 10.28%). The most represented categories among the cellular component category were “cell” (3,371, 18.03%), “cell part” (3,314, 17.72%), and “membrane” (3,313, 17.72%). In the molecular function category, the matched sequences were clustered into 12 classifications, and a majority of unigenes were divided into three subcategories “binding” (4,669, 45%), “catalytic activity” (3,830, 36.89%), and “transporter activity” (565, 5.44%). A total of 15,319 unigenes were divided into 25 functional classifications in the KOG database. The categories with the highest proportion of unigenes were “general function prediction only” (3,528, 23.03%) and “signal transduction mechanisms” (3,273, 21.37%). There were 18,705 unigenes significantly matched in the KEGG database and were assigned to 336 KEGG pathways involving six categories (cellular processes, environmental information processing, genetic information processing, human diseases, metabolism, and organismal systems). Among these pathways, the most enriched unigenes were “metabolic pathways” (2,516, 13.45%; ko01100), “pathways in cancer” (993, 5.31%; ko05200), and the “calcium signaling pathway” (782, 4.18%; ko04020). The KEGG classifications of unigenes are listed in Supplementary Figure 2.

### Functional Annotation of DEGs

**FIGURE 2:**
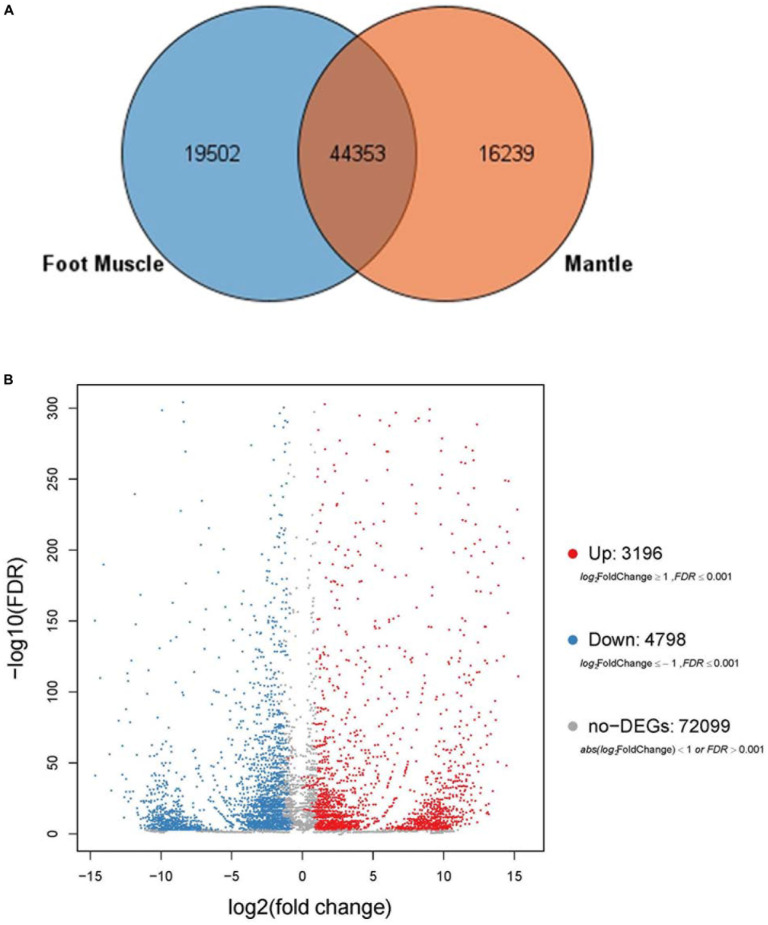
**(A)** Venn diagrams for number of comparisons of transcripts among foot muscle and mantle tissue. **(B)** Volcano plot of differentially expressed genes (DEGs) in foot muscle and mantle from *C. tritonis*.

Based on sequence similarities, a total of 44,353 unigenes were found to be conserved between the foot muscle and mantle transcriptomes (Figure 2A); 19,502 unigenes for foot muscle and 16,239 unigenes for mantle, specifically. Some of the differentially expressed unigenes may be responsible for the unique features of each of these species. A total of 7,994 DEGs (3,196 up-regulated and 4,798 down-regulated) were detected in *C. tritonis* foot muscle compared with mantle tissue (Figure 2B). The top 20 most highly expressed genes of the foot muscle and mantle are similar, as shown in Tables 2A,B, respectively. It is noteworthy that highly expressed unique sequences in the mantle were annotated as ferritin, which was probably involved in biomineralization.

**TABLE 2a:** (A) Most highly expressed genes in the foot muscle transcriptome.

Gene ID	FPKM	Expected count	Annotation	Species
CL5057.Contig3_All	11877.03	227556.58	NA	
Unigene1004_All	10901.33	290933.14	Cytochrome c oxidase subunit I (mitochondrion)	*Alcithoe lutea*
Unigene25450_All	6987.01	34236.72	NA	
Unigene965_All	6832.25	120549	NA	
Unigene29179_All	6022.21	39162	NA	
CL6630.Contig1_All	4850.91	8839	NA	
Unigene84857_All	4269.15	18026.34	Arylsulfatase B-like	*Mizuhopecten yessoensis*
Unigene2767_All	4073.99	24511	Metallothionein allelic variant 2	*Littorina littorea*
Unigene7119_All	3616.44	80508	Cytochrome b (mitochondrion)	*Columbella adansoni*
CL23.Contig3_All	3101.55	96749	PREDICTED: elongation factor 1-alpha	*Biomphalaria glabrata*
CL7409.Contig2_All	3067.15	78944.73	PREDICTED: filamin-A-like	*Aplysia californica*
CL23.Contig1_All	2813.56	94295.25	Hypothetical protein BRAFLDRAFT_122567	*Branchiostoma floridae*
CL1249.Contig3_All	2774.88	9660.64	Alpha-tubulin, partial	*Phyllonorycter ringoniella*
Unigene26238_All	2764.39	14780	NA	
CL2824.Contig1_All	2713.71	7266	PREDICTED: 40S ribosomal protein S25	*Aplysia californica*
Unigene66723_All	2640.42	19929	NA	
CL3723.Contig2_All	2517.48	40584	PREDICTED: actin, cytoplasmic	*Aplysia californica*
Unigene14502_All	2499.78	7827	Ribosomal protein rps15	*Lineus* viridis

**TABLE 2b:** (B) Most highly expressed genes in the mantle transcriptome.

Gene ID	FPKM	Expected count	Annotation	Species
Unigene28053_All	17863.36	90078.65	NA	
Unigene18554_All	10831.29	98693	NA	
Unigene1004_All	9724.1	266313.39	Cytochrome c oxidase subunit I (mitochondrion)	*Alcithoe lutea*
Unigene28567_All	9472.35	18235	NA	
Unigene19985_All	8244.64	27144	NA	
Unigene965_All	7155.5	129529	NA	
Unigene28951_All	6865.91	52161	Ferritin, partial	*Reishia clavigera*
Unigene29021_All	4124.78	22927	Uncharacterized protein LOC110239669	*Exaiptasia pallida*
CL23.Contig3_All	3989.31	127710	PREDICTED: elongation factor 1-alpha	*Biomphalaria glabrata*
CL23.Contig1_All	3658.3	125829.81	Hypothetical protein BRAFLDRAFT_122567	*Branchiostoma floridae*
Unigene8368_All	3590.27	13992	NA	
Unigene14502_All	3461.4	11085	Ribosomal protein rps15	*Lineus viridis*
Unigene7119_All	3276.67	74848	Cytochrome b (mitochondrion)	*Columbella adansoni*
CL2824.Contig1_All	3125.79	8556	PREDICTED: 40S ribosomal protein S25	*Aplysia californica*
Unigene13104_All	3005.92	8424	PREDICTED: ubiquitin-60S ribosomal protein L40	*Aplysia californica*
Unigene1937_All	2986.35	5125	60S Ribosomal Protein L37A	*Pantala flavescens*
CL7409.Contig2_All	2894.33	76446.85	PREDICTED: filamin-A-like	*Aplysia californica*
CL1249.Contig3_All	2831.93	10087.31	Alpha-tubulin, partial	*Phyllonory cterringoniella*
Unigene8670_All	2803.16	6600	PREDICTED: 60S ribosomal protein L35	*Condylura cristata*
Unigene8444_All	2713.35	9798	PREDICTED: 60S ribosomal protein L23a	*Aplysia californica*

**FIGURE 3:**
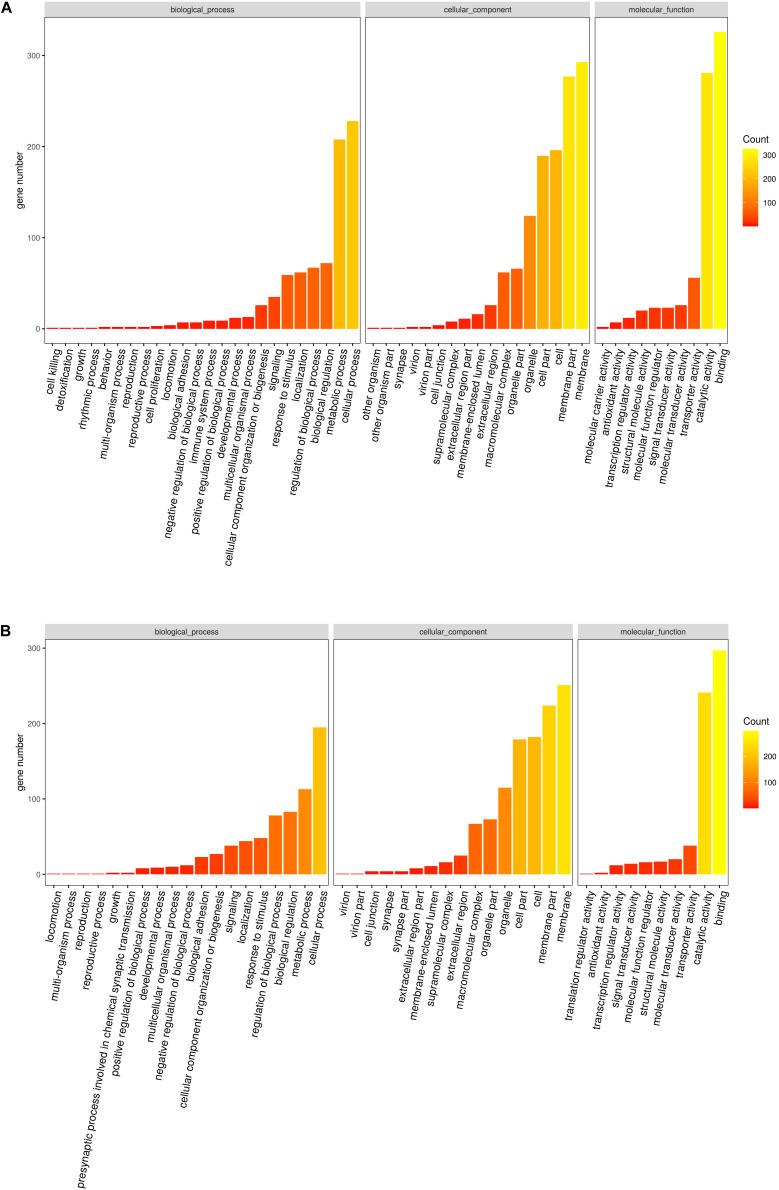
**(A)** Statistically common enriched Gene Ontology (GO) terms between foot muscle and mantle tissues for the up-regulated unigenes. **(B)** Statistically common enriched Gene Ontology (GO) terms between foot muscle and mantle tissues for the down-regulated unigenes.

Based on the gene expression level, GO enrichment analysis was performed to understand the functions of the exclusive DEGs in tissues and to search significantly enriched GO terms. All the DEGs were divided into three clusters categories (i.e., molecular biological function, cellular component, and biological process.). The vast majority of genes were involved in binding (623, 43.44%) and catalytic activity functions (522, 36.40%) in the molecular function category. In the cellular component class, most of the genes in the two tissues were related to the membrane (544, 22.25%), membrane part (501, 20.50%), and cell (378, 15.46%). In the biological process class, most genes were categorized as being related to cellular processes (423, 27.67%), metabolic processes (321, 21%), and biological regulation (155, 10.14%) (Supplementary Table 2). To investigate this further, GO-term enrichment was performed to gain insight into the potential functions of orthogroups shared exclusively between the two tissues using DEGs (Up-regulated and down-regulated genes) separately. The main functional classes of the listed up-regulated genes were consistent with the functional classification from down-regulated genes in GO categories (Figures 3A,B). Although similar compositions were observed for both tissues in the three major categories using GO assignments, there were some categories of biological processes that were differentially expressed between foot muscle and mantle, including GO terms linked to cell killing, detoxification, and molecular carrier activity. This finding indicates that some of the genes may be related to tissue-specific roles.

**FIGURE 4:**
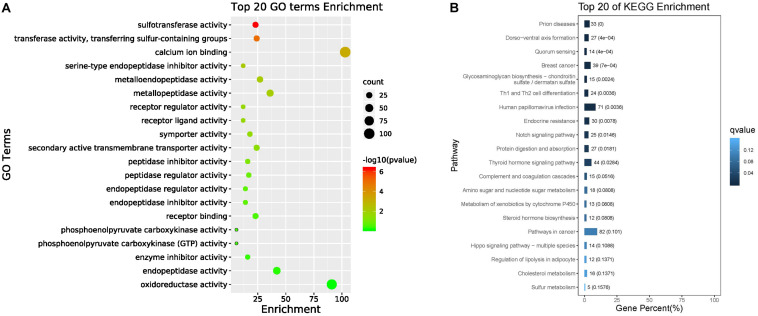
Top 20 enriched GO terms **(A)** and top 20 enriched KEGG pathways **(B)**.

The top 20 enriched GO terms related to DEGs in the molecular function category are listed in Figure 4A. The results revealed that some DEGs that were significantly enriched (FDR ≤ 0.01) in GO terms, including sulfotransferase activity, transferase activity, transferring sulfur-containing groups, and calcium ion binding, may be correlated with biomineralization. The up-regulated unigenes in the mantle were again mapped to reference pathways in the KEGG database based on function. KEGG pathway analysis indicated that DEGs significantly enriched in glycosaminoglycan biosynthesis-chondroitin sulfate/dermatan sulfate (ko00532, *p* = 0.000034), and sulfur metabolism (ko00920, *p* = 0.009006) may be related to biomineralization (Figure 4B).

### qPCR Validation of Genes Related to Biomineralization

**FIGURE 5:**
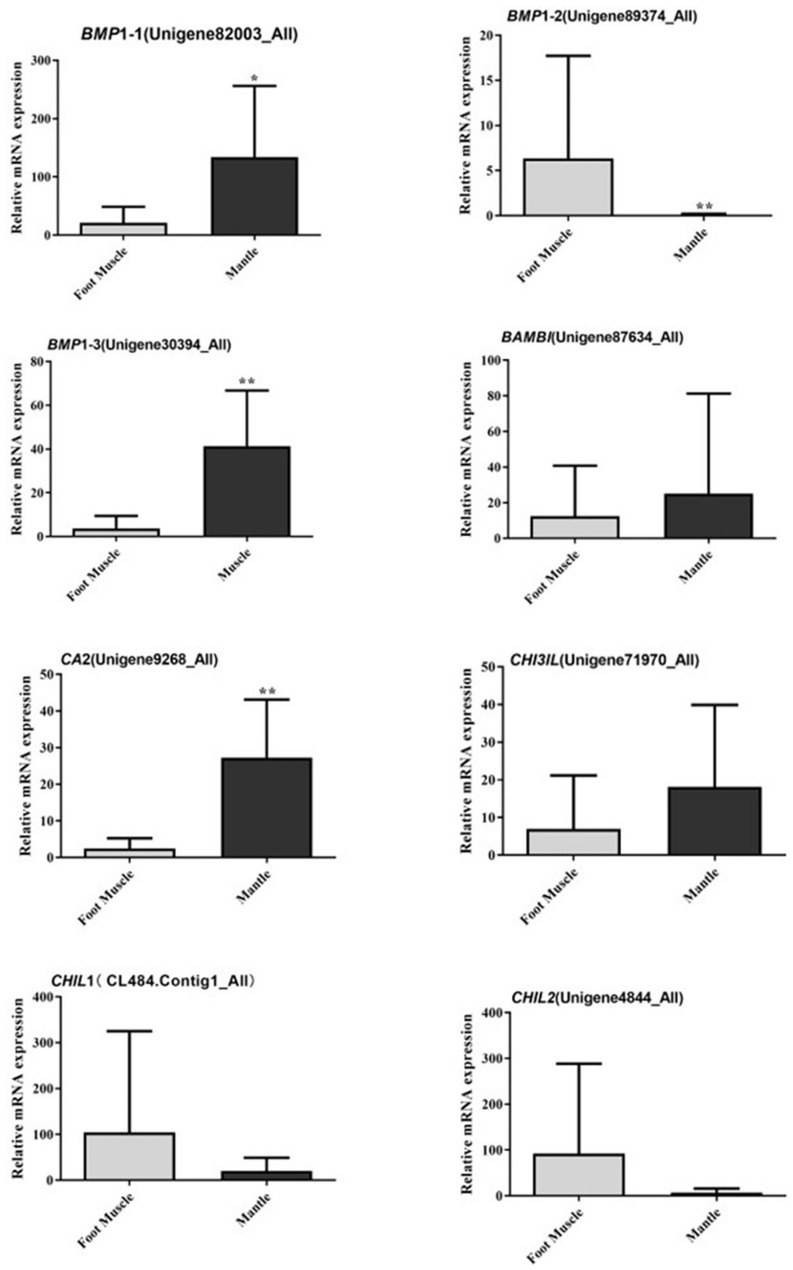
Relative mRNA expression profiles of eight selected biomineralization-related genes from mantle and foot muscle tissues of *C. tritonis*. [A *p*-value < 0.05 was considered significant (*), and *p* < 0.01 was considered highly significant (**)].

Twenty-four candidate sequences encoding members of the bone morphogenetic family, carbonic anhydrase, and chitinase were found in the transcriptome of *C. tritonis* as potentially involved in biomineralization. The biomineralization-related genes were examined by qPCR in the foot muscle and mantle (Table 3). Overall, bone morphogenetic protein 1 (Unigene82003_All), bone morphogenetic protein 1 homolog OS (Unigene30394_All), and carbonic anhydrase 2 OS (Unigene9268_All) were highly expressed in mantle tissue, whereas bone morphogenetic protein 1 (Unigene89374_All) was extremely significantly down-regulated in mantle according to qRT-PCR results, but a higher expression was found in transcriptome sequencing. Chitinase-3-like protein 1 (Unigene71970_All) was up-regulated in the mantle, while the other Chitinase-like lectin (CL484.Contig1_All, Unigene4844_All) showed lower expression in the mantle according to transcriptome sequencing qRT-PCR (Figure 5).

**TABLE 3:** Biomineralization-related DEGs examined by qPCR in mantle and foot muscle tissue.

Gene ID	Nr/Swissprot annotation	FDR	log2FC	Significant or not	qPCR validation
Unigene82003_All	Bone morphogenetic protein 1 [*Human*]	6.83E-12	5.564784619	Up, yes	Up (*P* > 0.05)
Unigene89374_All	Bone morphogenetic protein 1 [*Mouse*]	9.54E-05	8.912889336	Up, yes	Down (*P* > 0.01)
Unigene30394_All	Bone morphogenetic protein 1 homolog OS [*Strongylocentrotus purpuratus*]	2.71E-31	−8.997179481	Down, yes	Up (*P* < 0.01)
Unigene87634_All	K10162 BMP and activin membrane-bound inhibitor [*Mizuhopecten yessoensis*]	7.50E-07	3.967432138	Up, yes	Up (*P* > 0.05)
Unigene9268_All	Carbonic anhydrase 2 OS [*Tribolodon hakonensis*]	3.85E-09	1.930997212	Up, no	Up (*P* < 0.01)
Unigene71970_All	PREDICTED: chitinase-3-like protein 1 [*Aplysia californica*]	7.27E-271	12.033423	Up, yes	Up (*P* > 0.05)
CL484.Contig1_All	Chitinase-like lectin [*Littorina littorea*]	6.47E-123	3.230297619	Up, yes	Down (*P* > 0.05)
Unigene4844_All	Chitinase-like lectin [*Littorina littorea*]	7.24E-143	3.060460984	Up, yes	Down (*P* > 0.05)

### Phylogenetic Tree of Bone Morphogenetic Protein Gene Families

**FIGURE 6:**
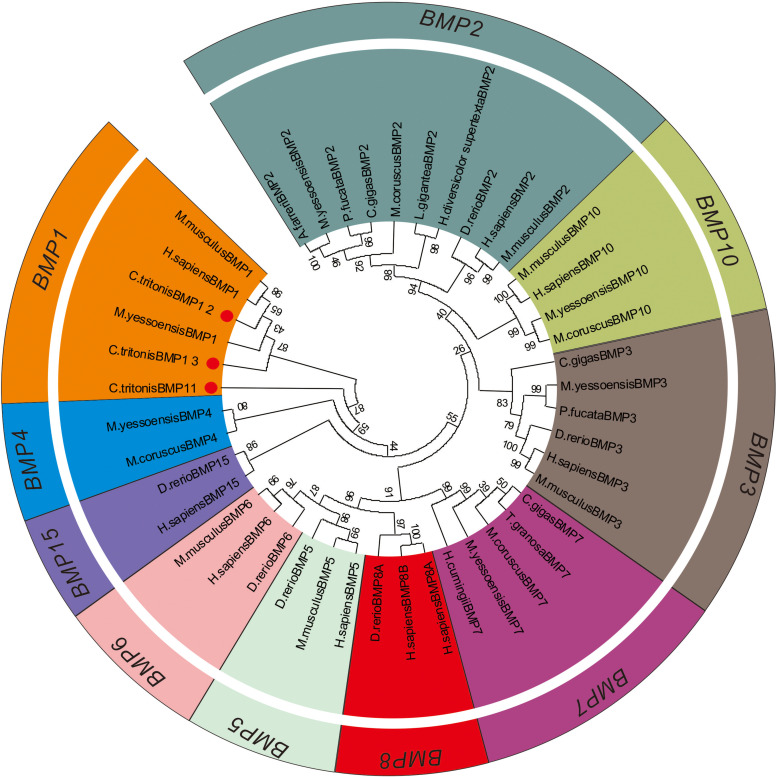
Phylogenetic tree of the bone morphogenetic protein (BMP) gene families.

A phylogenetic tree was constructed to identify the evolutionary history of *C. tritonis* bone morphogenetic proteins (BMP) based on deduced amino acid sequences of BMP from *C. tritonis* and other species (Supplementary Table 3). The results showed that three sequences of *C. tritonis* BMP (BMP1-1, BMP1-2, and BMP1-3) and BMP1 from other species were clustered into one branch, implying that *C. tritonis* BMP may be a category of the BMP1 subfamily. At the same time, BMP1-1 (Unigene82003_All) had a close genetic relationship with BMP1-3 (Unigene30394_All), but not with BMP1-2 (Unigene89374_All) in *C. tritonis* (Figure 6).

## Discussion

In this study, the comparative transcriptome of *C. tritonis* foot muscle and mantle were generated and assembled using the Illumina HiSeq sequencing platform. A total of 13.23 Gb bases were assembled into 109,722 unigenes (mean length = 640 bp), of which 46,239 were annotated, including 3,196 annotated significantly up-regulated genes and 4,798 significantly down-regulated genes in mantle [| log2(fold change)| ≥ 2 and FDR < 0.001]. Two reasons may explain the low-annotation rate: (1) At present, there is no research report on the genome sequencing of *C. tritonis*, and there is a lack of available *C. tritonis* transcriptome information in public databases. (2) The accuracy of next-generation sequencing technology greatly depends on the reference genome (Joubert et al., 2010; Kim et al., 2017; Lavelle et al., 2018).

According to the results of the GO terms enrichment analysis, in the molecular function ontology, most DEGs were enriched in sulfotransferase activity, transferase activity, transferring sulfur-containing groups, and calcium ion binding pathways. It is suggested that sulfotransferase catalyzes the transfer of sulfonic acid groups, which can affect the synthesis of chondroitin sulfate and then regulate biomineralization (Furuhashi et al., 2009; Sun et al., 2015; Zhang et al., 2020). KEGG enrichment analysis of the DEGs revealed major involvement in glycosaminoglycan biosynthesis-chondroitin sulfate/dermatan sulfate (ko00532), which performs biomineralization (Shi et al., 2013; Moreira et al., 2015; Li et al., 2017; Mao et al., 2018). Despite reports of many biomineralization-related genes in gastropods, very little is known about the basis of biomineralization in *C. tritonis* (Bootsma et al., 1988; Liu et al., 2011). Hence, we analyzed all of the unigenes known to be annotated with biomineralization.

Bone morphogenetic proteins are multi-functional growth factors that belong to the members of the transforming growth factor-beta (TGFβ) superfamily (Steiglitz et al., 2004; Cao and Chen, 2005; Boraldi et al., 2019). As is well known, BMPs are ubiquitous in a broad range of species and fulfill a variety of metabolic roles including osteogenesis. In the BMP family, BMP1 is a metalloprotease that plays an important role in the synthesis of collagen, the core substance of bones, by cleaving and activating lysyl oxidase (LOX) in the extracellular matrix, which promotes collagen cross-linking (Ge et al., 2006; Kokabu et al., 2012). In previous experiments, the knockout of BMP1 resulted in osteogenesis imperfecta in mice (Steiglitz et al., 2004; Maruhashi et al., 2010; Muir et al., 2014; Zhang et al., 2017). A phylogenetic analysis of BMP sequences between *C. tritonis* and other species was performed, and *C. tritonis* BMP1 were grouped in BMP1 clusters with other species, which suggested that *C. tritonis* BMP1 may belong to the BMP1 subfamily. At the same time, our data indicated that *C. tritonis* BMP1 genes displayed diverse expression patterns in different tissues, BMP1-1 (Unigene82003_All) and BMP1-3 (Unigene30394_All) genes were significantly up-regulated in the mantle compared with the foot muscle, indicating that BMPs play important roles in the shell formation. While BMP1-2 (Unigene89374_All) had a high expression in foot muscle tissue, suggesting a significant divergence between homologous genes in *C. tritonis*. The BMPs amino acid sequences clustering analysis of *C. tritonis* versus other species. A phylogenetic analysis revealed that *C. tritonis* BMP, in this study, created two separate clades: BMP1-1 had a close genetic relationship with BMP1-3, but not with BMP1-2, suggesting that the functions of these genes differentiated during evolution.

Carbonic anhydrase (CA) is believed to play crucial physiological roles in the biomineralization of mollusks due to their catalytic activity (Song et al., 2014; Le Roy et al., 2016; Cardoso et al., 2019; Zebral et al., 2019). In our analysis, CA was significantly up-regulated in the mantle, indicated that CA might participate in the mineralization of *C. tritonis*. This is the first report of BMPs and CA in *C. tritonis*, thus further study on BMPs and CA function needs to be undertaken in *C. tritonis*. Chitinase plays an important role in the metabolism of chitin, which is the major organic matrix protein of the shell, suggesting that chitinase may be involved in the mineralization of mollusks (Proespraiwong et al., 2010; Okada et al., 2013). Unlike in other mollusks, chitinase-related genes were not expressed at a higher level in the mantle. This distinct expression pattern suggested that chitin may play diverse roles in the foot muscle of *C. tritonis.* In previous studies, it has been revealed that ferritin, a transport protein ubiquitous in many organisms, was expressed exclusively in some tissues, especially the radula of marine mollusks, such as *Acanthopleura hirtosa, Patella laticostata*, and *Mytilus edulis*, and was considered to be potentially involved in biomineralization (Saunders et al., 2009; Narayanan et al., 2020). Ferritin was also found exclusively expressed in the mantle of *C. tritonis*, suggesting that it plays a similar role in biomineralization in C. tritonis. To our knowledge, this is the first time that biomineralization-related genes have been reported in *C. tritonis*. Overall, our analyses provide further insight into the biomineralization of *C. tritonis*.

This study is the first to represent transcriptomes of mantle and foot muscle tissues from *C. tritonis*, which provides transcriptomic resources for future genetic or genomic studies on this mollusk. Transcriptome comparison assists in better understanding of the molecular mechanisms of biomineralization in *C. tritonis.*

## Data Availability Statement

The datasets presented in this study can be found in online repositories. The names of the repository/repositories and accession number(s) can be found below: NCBI (accession: PRJNA695322).

## Author Contributions

GZ: experiment, writing, and editing. WL: reviewing. HZ: supervision. MH: project administration and approval. All authors contributed to the article and approved the submitted version.

## Conflict of Interest

The authors declare that the research was conducted in the absence of any commercial or financial relationships that could be construed as a potential conflict of interest.

## Footnotes

1 http://blast.ncbi.nlm.nih.gov/Blast.cgi

2 www.PremierBiosoft.com
