# Supplementary material for: Comparative Transcriptomic and Expression Profiles Between the Foot Muscle and Mantle Tissues in the Giant Triton Snail Charonia tritonis
Source: Front Physiol. 2021 Feb 24;12:632518. doi: 10.3389/fphys.2021.632518 (PMC7959727; doi:10.3389/fphys.2021.632518)
Supplement: Supplementary file 1 [file Data_Sheet_1.ZIP › Supplementary Tables.docx]

Supplementary Material

## Supplementary Tables

**Supplementary Table 1 List of primers used for quantitative RT-PCR validation**

| Gene symbol | | Gene ID |  | Primer sequence (5′to 3′) | | |  |
| --- | --- | --- | --- | --- | --- | --- | --- |
|  |  |  |  |  |  |  |  |
| BMP1-1 |  | Unigene82003_All | | F:GCCACCACCATCTCTACTTCT | | | |
|  |  |  |  | R:GACAATAGGCTTGAGGTAGGAAC | | | |
| BMP1-2 |  | Unigene89374_All | | F:AATACAACCGCAGACCAAGTG | | | |
|  |  |  |  | R:CTTAGCCACCAGCAATGTTCT | | | |
| BMP1-3 |  | Unigene30394_All | | F:TGTGCTGCTAGTGTCGGAAG | | |  |
|  |  |  |  | R:GCTGTATCCTCCTGGCTTGAA | | | |
| BAMBI |  | Unigene87634_All | | F:TCTGCTCGCTCTCGTCATC | | |  |
|  |  |  |  | R:GGTGGTCGTGATAGTAGTGGTA | | | |
| CA2 |  | Unigene9268_All | | F:GGTCATTAAGGTGCGAAGGAA | | | |
|  |  |  |  | R:CTGAACAGATGAAGGCATTACG | | | |
| CHI3L1 |  | Unigene71970_All | | F:TCAGCAAGAGTGTGGAGAAGG | | | |
|  |  |  |  | R:GACAGTAGTAGCGGCACCAT | | | |
| CHIL1 |  | CL484.Contig1_All | | F:CGGATGGTTCTTATGCTGAT | | |  |
|  |  |  |  | R:CGTTCTCACACTTCACCTT | | |  |
| CHIL2 |  | Unigene4844_All | | F:AACTTCCTCCTCCACCTCCT | | |  |
|  |  |  |  | R:CGCACACCACATCACATTCA | | | |
| 18S |  |  |  | F:ATGGTCAGAACTACGACGGTAT | | | |
|  |  |  |  | R:GTATTGCGGTGTTAGAGGTGAA | | | |

## Supplementary Table 2 Functional annotation of DEGs in the GO database

| Item | Function group | Number of unigenes | Item | Function group | Number of unigenes |
| --- | --- | --- | --- | --- | --- |
| Biological_process | Behavior  Biological adhesion  Biological regulation  Cell killing  Cell proliferation  Cellular component or-  ganization or biogenesis  Cellular process  Detoxification  Developmental process  Growth  Immune system process  Localization  Locomotion  Metabolic process  Multi-organism process  Multicellular organismal- process  Negative regulation of-  biological process  Positive regulation of-  biological process  Presynaptic process involved-  in chemical synaptic- transmission  Regulation of biological-  process  Reproduction  Reproductive process  Response to stimulus  Rhythmic process | 2  30  155  1  3  53  423  1  21  3  9  106  5  321  3  23  19  17  2  145  3  3  107  1 | Biological_process  Cellular_component  Molecular_function | Signaling  Cell  Cell junction  Cell part  Extracellular region  Extracellular region part  Macromolecular complex  Membrane  Membrane part  Membrane-enclosed lumen  Organelle  Organelle part  Other organism  Other organism part  Supramolecular complex`  Synapse  Synapse part  Virion  Virion part  Antioxidant activity  Binding  Catalytic activity  Molecular carrier activity  Molecular function regulator  Molecular transducer activity  Signal transducer activity  Structural molecule activity  Transcription regulator- activity  Translation regulator activity  Transporter activity | 73  378  8  369  51  19  129  544  501  27  239  139  1  1  24  5  4  3  3  9  623  522  2  39  46  37  37  24  1  94 |

**Supplementary Table 3** Phylogenetic tree construction of different types of bone morphogenetic proteins (BMPs)

| **Species Name** | **Abbreviated** | **Bone morphogenetic protein** | **Uniprot Entry** |
| --- | --- | --- | --- |
| *Homo sapiens* | H.s | BMP1 | P13497 |
|  |  | BMP2 | P12643 |
|  |  | BMP3 | P12645 |
|  |  | BMP5 | P22003 |
|  |  | BMP6 | P22004 |
|  |  | BMP8a | Q7Z5Y6 |
|  |  | BMP8b | P34820 |
|  |  | BMP10 | O95393 |
|  |  | BMP15 | O95972 |
| *Mus musculus* | M.m | BMP1 | P98063 |
|  |  | BMP2 | P21274 |
|  |  | BMP3 | Q8BHE5 |
|  |  | BMP5 | P49003 |
|  |  | BMP6 | P20722 |
|  |  | BMP10 | B7ZN66 |
| *Danio rerio* | D.r | BMP2 | O13109 |
|  |  | BMP3 | A0JML2 |
|  |  | BMP5 | Q7T288 |
|  |  | BMP6 | F1R678 |
|  |  | BMP8 | Q1LWW7 |
|  |  | BMP15 | Q0VG66 |
| *Mizuhopecten yessoensis* | M.y | BMP1 | A0A210QHK7 |
|  |  | BMP2 | A0A210PQ13 |
|  |  | BMP3 | A0A210PF98 |
|  |  | BMP4 | A0A210PNT3 |
|  |  | BMP7 | A0A210PQS7 |
|  |  | BMP10 | A0A210QNM3 |
| *Mytilus coruscus* | M.c | BMP2 | A0A6J8DZE7 |
|  |  | BMP4 | A0A6J8E3C7 |
|  |  | BMP7 | A0A6J8E5J4 |
|  |  | BMP10 | A0A6J8ETM6 |
| *Crassostrea gigas* | C.g | BMP2 | K1PQP9 |
|  |  | BMP3 | K1R9I6 |
|  |  | BMP7 | K1RIZ3 |
| *Hyriopsis cumingii* | H.c | BMP7 | A0A161AT82 |
| *Tegillarca granosa* | T.g | BMP7 | J7G3X1 |
| *Lottia gigantea* | L.g | BMP2 | A0A0B6VJK3 |
| *Pinctada fucata* | P.f | BMP2 | Q75NB6 |
|  |  | BMP3 | A0A191Z434 |
| *Haliotis diversicolor supertexta* | H.d | BMP2 | A0A3S7T0A6 |
| *Azumapecten farreri* | A.f | BMP2 | A0A0U1UKT7 |
