# Supplementary material for: Comparative Transcriptomic and Expression Profiles Between the Foot Muscle and Mantle Tissues in the Giant Triton Snail Charonia tritonis
Source: Front Physiol. 2021 Feb 24;12:632518. doi: 10.3389/fphys.2021.632518 (PMC7959727; doi:10.3389/fphys.2021.632518)
Supplement: Supplementary file 2 [file Data_Sheet_2.ZIP › Supplementary Figures.docx]

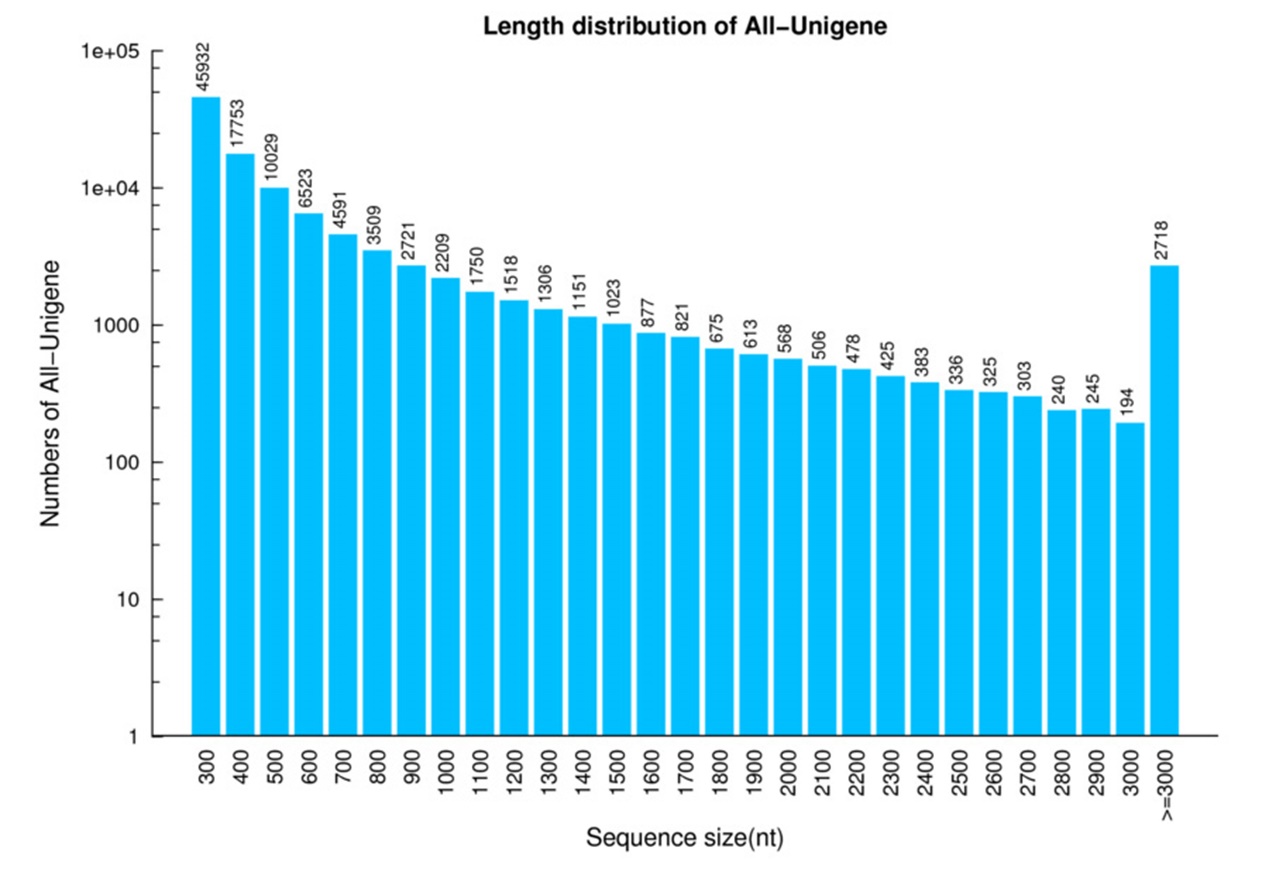


**Supplementary Figure S1.** Length distribution of All-Unigenes


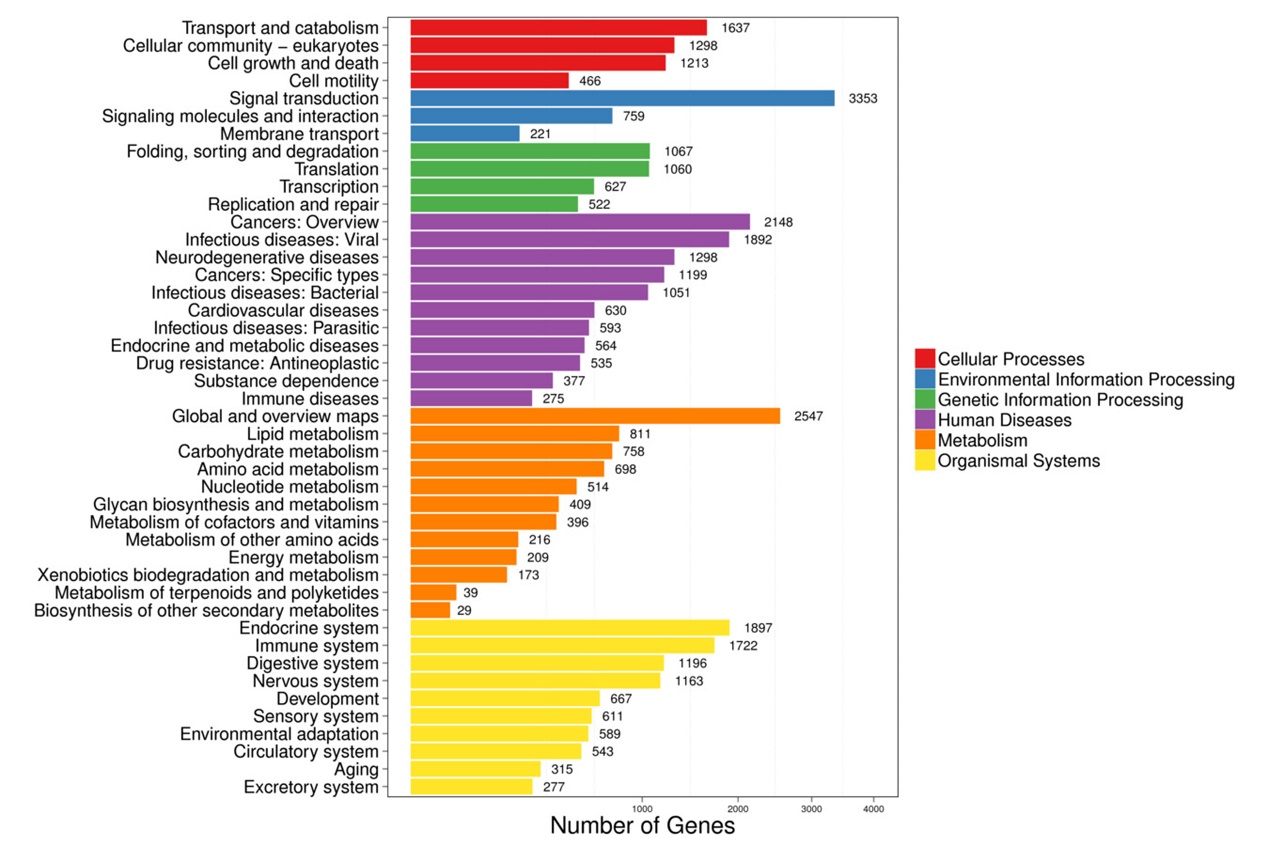


**Supplementary Figure S2.** Functional distribution of KEGG annotations.
